# State-of-the-Art Optical Microfiber Coupler Sensors for Physical and Biochemical Sensing Applications

**DOI:** 10.3390/bios10110179

**Published:** 2020-11-18

**Authors:** Maolin Dai, Zhenmin Chen, Yuanfang Zhao, Manthangal Sivanesan Aruna Gandhi, Qian Li, Hongyan Fu

**Affiliations:** 1 Tsinghua-Berkeley Shenzhen Institute, Tsinghua University, Shenzhen 518055, China; dml19@mails.tsinghua.edu.cn (M.D.); chenzhm@pcl.ac.cn (Z.C.); zhaoyf19@mails.tsinghua.edu.cn (Y.Z.); 2 School of Electronic and Computer Engineering, Peking University, Shenzhen 518055, China; aruna@pkusz.edu.cn (M.S.A.G.); liqian@pkusz.edu.cn (Q.L.)

**Keywords:** optical fiber, microfiber coupler, sensors, physical sensors, chemical sensors, biosensors

## Abstract

An optical fiber coupler is a simple and fundamental component for fiber optic technologies that works by reducing the fiber diameter to hundred nanometers or several micrometers. The microfiber coupler (MFC) has regained interest in optical fiber sensing in recent years. The subwavelength diameter rationales vast refractive index (RI) contrast between microfiber “core” and surrounding “cladding”, a large portion of energy transmits in the form of an evanescent wave over the fiber surface that determines the MFC ultrasensitive to local environmental changes. Consequently, MFC has the potential to develop as a sensor. With the merits of easy fabrication, low cost and compact size, numerous researches have been carried out on different microfiber coupler configurations for various sensing applications, such as refractive index (RI), temperature, humidity, magnetic field, gas, biomolecule, and so on. In this manuscript, the fabrication and operation principle of an MFC are elaborated and recent advances of MFC-based sensors for scientific and technological applications are comprehensively reviewed.

## 1. Introduction

In recent years, the emerging trends of optical sensing technology have elicited inventive sensors for various industrial application scenarios namely aerospace, infrastructure, transportation, biochemistry, and so on. In the rapid progress of sensors’ establishment, optical fiber sensors (OFS) have played an aggregately indispensable role; hitherto, the first optical fiber with low attenuation was fabricated by Corning and has the merits of being light in weight, compact in size, and low in cost, as well as possessing electromagnetic immunity and excellent security.

In the last few decades, numerous optical fiber technologies have been developed, such as fiber Bragg grating (FBG) [1,2,3], long-period grating (LPG) assisted fiber [4,5], photonic crystal fiber (PCF) [6,7], and multicore fiber (MCF) [8,9] with surface plasmon resonance (SPR) [10,11,12], to achieve the different requirements of point-type optical fiber sensing. However, the relatively complex fabrication, high cost, and relatively low sensitivity limit their applications. Microfiber, first reported by Tong et al. in 2003, with a diameter confined to tens of nanometers to several micrometers [13], has been intensively investigated in fiber optics as a combination of fiber optics and nanotechnology. Typically, microfiber has a diameter of hundreds of nanometers to several micrometers, with excellent diameter uniformity and sidewall smoothness [14]. When the diameter of microfiber reaches subwavelength scale, the light diffraction limit causes the penetration of a large portion of evanescent field into the environment, which enhances the light–matter interaction, thanks to the highly sensitive signal that occurs at the ambient surroundings, to investigate the sensing performances. Besides, microfiber-based devices are promising to be integrated on a chip, in line with the trend of future development of integrated silicon photonics. Benefiting from the flexibility of microfiber, different structures based on it, namely microfiber knot [15,16], loop [17,18], coil [19,20], and microfiber coupler (MFC), are investigated to be excellent sensing elements.

As a basic and common component of fiber optics, optical fiber coupler has been studied intensively since the 1980s [21,22]. It is also identified as an optical splitter, with a function to split and recombine light signal in different fibers, and is critical to fiber optic communication. However, it has received much attention in the sensing field in recent years by reducing the diameter to micro/nanoscale with the development of nanotechnology. MFCs with a uniform waist region and two transition regions are usually fabricated by heating and pulling two standard single-mode fibers together and simultaneously. The two fibers penetrate and stick to one another, in the waist region, under the high temperature. Additionally, MFC tip [23,24] and MFC loop [25,26,27,28,29,30,31,32] are made by cutting off the waist region and connecting the two output ports, respectively. The two MFC-based structures are reported as having potential in the sensing area with their advantages. For example, the MFC tip was used to detect the analytes through infinitesimal insertion, similarly to a probe. For an MFC loop, the input and output are at one side like an MFC tip, and the light signal has reflected by the Sagnac loop. Based on this, the MFC loop is easy to be integrated with other sensing elements to realize multiparameter simultaneous measurement. The subwavelength diameter of the coupler waist region results in a large portion of evanescent field, which offers the incubator for mode coupling of the odd supermode and even supermode. The modal interference between the odd supermode and even supermode has to owe their different propagation constants and is easily affected by surroundings. In 2009, Jung et al. reported the MFC for broadband (400~1700 nm) single mode operation due to the high-order filtering characteristics, pointed out that MFC was possible to utilize in sensing applications [33]. After that, numerous physical, chemical, and biosensors based on MFC have been demonstrated. Refractive index (RI) [25,28,29,34,35,36,37,38,39,40,41,42,43,44,45,46,47,48,49], force [50], twist [27,51], vibration [52], and strain [53] have direct effect on the signal transmission of MFC waist region. By covering and well packaging the waist region with functional materials, monitoring of temperature [23,24,28,31,32,37,42,43,46,54,55,56,57,58,59,60], humidity [31,57,61,62,63,64,65,66], magnetic field [30,67,68,69], flow velocity [70], gas [62,71,72,73,74] and molecule concentration [49,75,76,77,78,79,80,81] with advantages of high sensitivity, real time, and quick response may perhaps be achieved. We summarize the important works for wide applications in Table 1, to show the rapid development of MFC-based sensors.

In this paper, first and foremost, we have briefly introduced the MFC-based sensors and further systematically review the sensing principle, fabrication, state of the art and prospects of the technologies. The technical background and introduction are presented in Section 1; fundamental and fabrication methods of MFC are corroborated in Section 2; the recent progress in MFC-based sensors for different applications are discussed and summarized in Section 3; finally, yet importantly, we conclude and provide the prospects and challenges in Section 4.

## 2. Fabrication and Operation Principle

In this section, fabrication methods of MFC and the sensing operation principle of MFC-based sensors are elaborated upon. Typically, there are three major fabrication methods to build an MFC: side polishing method [82,83], chemical etching method [34,84,85], and heating-and-pulling method [36,58]. Therein, the heating-and-pulling method has been widely used for making an MFC with the advantages of low cost, simple operation, precise control, and environmental non-toxic. The fabricated MFC has a uniform waist and adjustable geometrical shape. For sensing applications, the subwavelength diameter of MFC causes a high RI contrast between the sensor and ambient environment, thus leading to a large evanescent field on the fiber surface, which is influenced easily by surrounding changes. Furthermore, engineering of the geometric size of MFC also affects the coupling between two identical microfibers.

### 2.1. Fabrication

**Figure 1:**
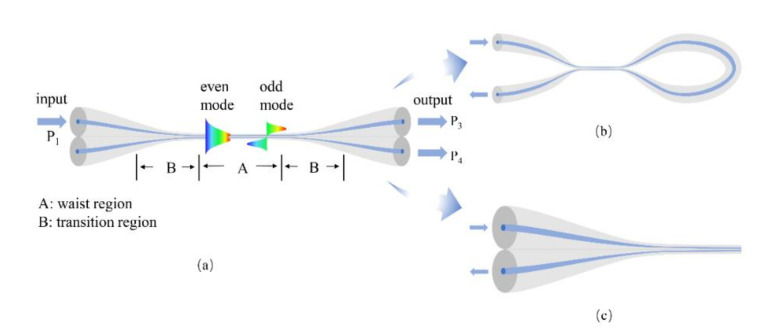
The schematic of (**a**) MFC, (**b**) MFC loop, and (**c**) MFC tip. The waist region and transition region are mentioned in (**a**).

In general, the MFC is made by single mode fiber (SMF) and consists of four input/output ports, with two symmetrical transition regions and a uniform waist region. Based on this, to cleave the waist region or connect two input/output ports on the same side, an MFC tip and MFC loop are engineered. The schematics of the three structures are shown in Figure 1. There are mainly three methods to fabricate an MFC: side polishing method [82,83], chemical etching method [34,84,85], and heating-and-pulling method [36,58]. Side polishing method is to taper the fibers by lateral grinding the fiber cladding and then putting the two polished microfibers together, to form an MFC. Due to the disadvantages of difficult fabrication and low yield rate, this method is rarely used for current researches. Chemical etching method has based on the principle that silica reacts with Hydrofluoric (HF) acid. In this method, MFC could be packaged immediately after being etched in the cuvette, which is convenient for the next experiments. The etching degree may be controlled by accurately modulating the etching time, but the processing time is relatively long. However, HF is toxic to the human body.

The heating-and-pulling method is widely used in the fabrication of various devices related to microfiber owing to the virtues of low loss, good stability, and excellent repeatability. Fabricated by this method, two standard optical fibers are twisted slightly with each other. The ends of microfibers are fixed on two translational motorizing stages, and the twisted region suspends in the air. A micro-heater is utilized to heat the twisted region and makes it softened and able to be stretched and elongated. Meanwhile, the two mobile stages move reversely towards each other, and the micro-heater sweeps back and forth simultaneously. The entire process is controlled by a stepper motor and a computer with control software. An ideal MFC is developed by carefully controlling the heating temperature, flame flow, sweeping of micro-heater, and moving of the translational stages. In the fabrication process, the optical fibers are tapered in an adiabatic way, which means the fundamental mode in the fibers may be mostly converted into a guided mode in the MFC [86].

### 2.2. Operation Principle

Depending on the fusion degree, MFCs may be classified into weakly fused MFCs and strongly fused MFCs [87]. For weakly fused MFCs, the two identical microfibers roughly maintain their original cross-section geometries; they possess dumbbell-shaped cross sections. In this case, the aspect ratio, which is defined as the ratio between waist width and height of the cross-section at the waist region of MFC, is usually larger than 1.8. However, strongly fused MFCs have an aspect ratio that varies from 1.0 to 1.8 [29,88]. In practical, strongly fused MFCs have elliptical- or circular-shaped cross-sections, the coupling lengths are longer than that of weakly fused MFCs, and critical diameters are larger compared with weakly fused MFCs. Moreover, strongly fused MFCs show weaker dependence on polarization and external RI change than weakly fused MFCs. As a result, weakly fused MFCs are more appropriate to develop as sensors [87,88,89,90].

The coupling coefficients, $C_{w}$ for weakly fused MFCs and $C_{s}$ for strongly fused MFCs, can be expressed by the following equations [66]:

(1) $$\left\{\begin{array}{c} C_{w}=\frac{2k}{r}\cdot {\left(\frac{\Delta }{2\pi D}\right)}^{1/2}\cdot \frac{U_{\infty }}{V^{5/2}e^{V\left(2D-2\right)}}\\ C_{s}=\frac{3\pi \lambda }{32n_{eff}r^{2}}\cdot \frac{1}{{\left(1+1/V\right)}^{2}} \end{array}\;,\right.$$

where k is a constant, $\Delta \;=\left(n_{eff}{}^{2}-n_{3}{}^{2}\right)/2n_{eff}{}^{2}$ is the relative RI difference, and $V=2\pi r{\left(n_{eff}{}^{2}-n_{3}{}^{2}\right)}^{1/2}/\lambda$ refers to normalized frequency. Moreover, $n_{eff}$ and $n_{3}$ are the effective RI of microfiber and the RI of surrounding medium, respectively, r is the radius of microfiber, d is the inter-core distance between two microfibers, and D=d/2r describes the fusion degree. $U_{\infty }=2.405$ is the limiting value of the normalized frequency to ensure the single-mode transmission. λ is the wavelength of incident light. The output power, P, of the coupled port is given by the following:

(2) $$P=P_{0}\sin^{2}\left(CL\right),$$

where C is the coupling coefficient, L is the effective coupling length, and $P_{0}$ is the input power.

For weakly coupled MFCs, according to the supermode theory [91], the two microfibers with subwavelength scale diameters are regarded as a new waveguide. The fiber core and cladding are fused to form a uniform medium, which acts as a new “core”; 0 to support the transmission of the incident light, the external medium acts as a new “cladding”. When the light of TE/TM polarization with power $P_{1}$ is launched into port 1, the simultaneous excitation of both odd supermode and even supermode happens; supermodes transmit along the direction of length, and coupling with each other to oscillate energy. The power $P_{3}$ in port 3 and $P_{4}$ in port 4 satisfy the following equations [91]:

(3) $$P_{3}=P_{1}\cos^{2}\frac{\varphi }{2},$$

(4) $$P_{4}=P_{1}\sin^{2}\frac{\varphi }{2},$$

where φ is the phase difference of the two supermodes accumulated along with the coupling region. The phase difference has achieved an extreme in the uniform waist region and is less affected by the two transition regions, owing to the enormous variation of geometric sizes. Therefore, we can get the phase difference, φ, as follows [91]:

(5) $$\varphi =\frac{2\pi L\left(n_{eff}^{even}-n_{eff}^{odd}\right)}{\lambda },$$

where $n_{eff}^{even}$ and $n_{eff}^{odd}$ are the effective refractive indices of the even and odd supermodes; L is the coupling length, namely the length of the waist region; and λ is the wavelength of incident light. The location of the wavelength dip, $\lambda_{N}$, in the transmission spectrum satisfies the following equation [39]:

(6) $$\varphi_{N}=\frac{2\pi L\left(n_{eff}^{even}-n_{eff}^{odd}\right)}{\lambda_{N}}=\left(2N-1\right)\pi ,$$

where $\varphi_{N}$ represents the phase difference of the $N_{th}$ wavelength dip, and *N* is a positive integer. From the equations above, the modal interference depends on both the waist region and effective refractive indices of the supermodes, which are affected by ambient medium easily. Based on this, MFCs have been widely explored as highly sensitive physical sensors, chemical sensors, and biosensors. When the MFCs are developed as sensors to detect ambient changes or biomolecule, both the intensity modulation [24,68] and wavelength modulation [23,55] can be employed to estimate the environmental parameters. However, the intensity modulation may be affected by the stability of sensing system, such as the performance of light source or ambient disturbance. As a consequence, tracking of wavelength shift is more suitable for high-performance sensing applications, which is used in most MFC-sensors investigations.

## 3. Sensing Applications

Since Jung et al. reported the MFC for broadband single mode operation and its sensing application potential due to the high-order filtering characteristics [33], the MFC-based sensor has become a subject of intensive research in recent years, owing to its simple fabrication, compact size, ultrahigh sensitivity, fast response, and convenience for on-chip integration. Apropos of other optical fiber structures, the MFC is a novel sensitive element to realize sensing application. By cleaving off the waist region, the MFC tip plays a role as a sensor probe. The transmission light is reflected by the microfiber facet due to the Fresnel effect, which is the potential to detect the analytes through a tiny point of insertion. A Sagnac loop has formed when connecting the two output ports. The light signal passes through the MFC and is reflected by the Sagnac mirror. It is of great potential to realize multiple parameters’ simultaneous measurement by combining the loop with other fiber-based sensing elements, including polarization-maintaining fiber (PMF)-based sensors. The applications of the three structures (MFC, MFC loop, and MFC tip) for sensors are utilized to online monitor physical and biochemical parameters.

### 3.1. Physical Sensors

The MFC with micro/nanoscale has a largely evanescent field which is highly sensitive to external RI variation. Temperature-, magnetic-field-, and humidity-sensing may be implemented with transducers such as thermo-optic materials, magnetic fluid (MF), and hydrophilic materials. Strain, twist, and vibration can be directly detected by MFC, since the geometric parameters of MFC are tuned by them and influence the coupling state of two supermodes. For the detection of other physical parameters, the well-designed sensing system has necessitated. For example, for the flow-rate sensing, the researchers combined a helical MFC with a gold-coated glass capillary, to realize a “hot-wire” microfluidic flowmeter [70].

#### 3.1.1. RI Sensors

The MFC can perhaps be directly exposed to air or liquid for detecting the RI variation. From the previous studies, the sensitivity of RI sensing ranges from 10^3^ to 10^6^ nm/RIU, which evidences a high dependence on ambient RI change. In 2007, Hidehisa Tazawa et al. proposed an MFC for RI sensing [49]. An RI variation of 4 × 10^−6^ RIU has been evaluated to be a noise-equivalent level perhaps detected. In 2011, an MFC sensor was fabricated by a two-step method to realize RI sensing with a sensitivity of 1125 nm/RIU [34]. In the experiment, the diameter of the coupler is 30–35 μm after heating and pulling, then HF solution (HF 20 wt% in water) has been used to further decrease the coupler diameter. To enhance the mechanical strength of the coupler, a silica rod of 40 mm length was employed to pack and protect the coupler. Additionally, the authors corroborate that the MFC with a smaller diameter induced by a longer etching time will assist the RI sensor to enhance the sensing performances.

Although the diameter of MFC may be well controlled by adjusting the etching time, the process is relatively complicated and time-consuming. Most of the studies use heating-and-pulling method to make an MFC. Bo et al. reported a 2.5 μm-diameter MFC RI sensor [36], with an average sensitivity of 2723 nm/RIU in the RI range from 1.3340 to 1.3800, which covers the generic biological range. Furthermore, the sensor presents high sensitivity of 4155 nm/RIU in the analyte RI environment from 1.3340 to 1.3515. The RI sensing performances of MFC tip and MFC loop have further investigated. The MFC tip with a diameter of 2.1 μm exhibits a sensitivity of 6142.0 nm/RIU within the range of 1.39–1.40 [41], and the microfiber loop with a diameter of 4.5 μm facilitates a sensitivity of 3617 nm/RIU in the analyte RI range of 1.33–1.41 [29]. It is proved that the sensitivity versus external RI increases when the fiber diameter is getting smaller. However, the diameter should be larger than the odd mode cutoff value to ensure the supermode interference. When the diameter becomes smaller, the fragility of structure and dynamic range of detection play a negative role for RI sensing. Besides, tip and loop structures have their advantages. For the tip structure, the end of the MFC acts as a reflection-based probe to get access to samples through a tiny point of insertion [24]. The loop structure also makes the input and output at the same end, which is convenient for practical implementation and is easy to realize multiparameter coexistent monitoring by combining with other sensing elements [32].

**Figure 2:**
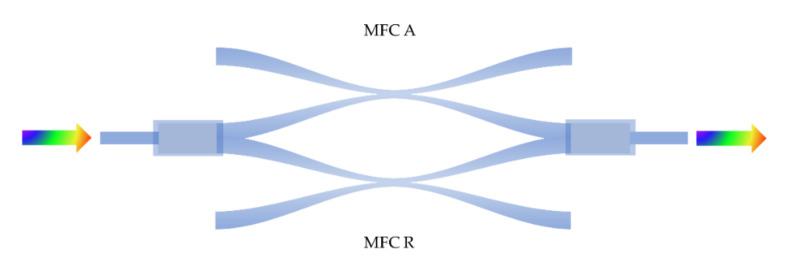
The schematic diagram of Mach–Zehnder interferometer (MZI) made by two MFCs.

In recent years, the sensing performance of MFC has been improved a lot by utilizing novel mechanisms. For example, researchers enhance the sensing sensitivity by introducing birefringence induced the Vernier effect into the sensing mechanism [25,48,80]. Concerning Reference [47], the Vernier effect was realized by using two MFCs to form a Mach–Zehnder interferometer (MZI); schematic diagram of MZI is exhibited in Figure 2.

The coupler *R* and coupler *A* act as the reference arm and sensing arm of the MZI, respectively. The phase difference between coupler *A* and coupler *R* is induced by their slightly different structural parameters. For the transmission signal, the power satisfies the following equation [47]:

(7) $$P_{out}=P_{A}+P_{R}+2\sqrt{P_{A}\cdot P_{R}}\cos\left(\Delta \varphi \right),$$

where Δφ is the phase difference between the light in coupler R and light in coupler A. By forming an MZI, the small RI variation outside of the coupler A would lead to a large dip wavelength shift in output envelope spectrum; therefore, the sensitivity is greatly enhanced. The RI sensing experiments show that the MFC-based sensor with Vernier effect possesses a sensitivity of 126,540 nm/RIU, which is about 20 times better than the single coupler without the Vernier effect.

Utilizing the unique characteristics of MFC can also enhance the sensing performance of MFC-based sensors. Wanvisa et al. [46] reported an MFC interferometer with a fiber diameter of 560 nm. By tracking the spectral dips near the odd mode cutoff region, an ultrahigh sensitivity of 480,000 nm/RIU was achieved, which is the highest record, to the best of our knowledge. On the one hand, the ultrahigh sensitivity is beneficial to further improve the detection limit; on the other hand, it can reduce the dynamic range owing to the restriction of instrument detection range.

The spectral dependency on the external RI S is obtained as follows [46]:

(8) $$S=\frac{\partial \lambda_{N}}{\partial n}=\frac{\lambda_{N}}{\Delta n_{eff}-\frac{\lambda_{N}\partial \left(\Delta n_{eff}\right)}{\partial \lambda }}\frac{\partial \left(\Delta n_{eff}\right)}{\partial n},$$

where $\Delta n_{eff}$ represents the effective RI difference between two supermodes, and $G=\Delta n_{eff}-\lambda_{N}\partial \left(\Delta n_{eff}\right)/\partial \lambda$ is defined as the effective group index difference between two supermodes. It is clear that when G approaches 0, the sensitivity would reach infinite. The spectral dips move in the opposite direction on either side of the point where the effective group index difference equals to 0, particularly at the dispersion turning point. Utilizing this principle, Li et al. reported an MFC sensor with a diameter of 1.8 μm [42]. Sensitivities of 59,624 and −58,470 nm/RIU around the surrounding RI of 1.333, and 26,999 and −29,267 nm/RIU around the surrounding RI of 1.35, have been obtained, respectively.

MFC is sensitive to ambient RI change, owing to the subwavelength diameter, as reported by numerous. The smaller diameter is, but necessary larger than the odd supermode cutoff diameter, the higher sensitivity perhaps obtained. Enhancement of sensitivity might be realized by different methods, but we should trade off the robustness, sensitivity, dynamic range, and other considerations for different sensing objects.

#### 3.1.2. Temperature Sensors

Due to the limitation of the thermo-optic coefficient and thermo-expand coefficient of silica, MFC is less sensitive to surrounding temperature change. Assisting temperature-sensitive materials are applied to enhance the response of MFC for temperature-sensing applications. We first summarize the previous MFC-based temperature sensors with different materials in Table 2; we then discuss their performance in detail.

When ambient temperature changes, the thermo-optic effect and thermo-expand effect would induce an RI change and a length change on the waist of MFC, thus influence the coupling status of two supermodes. The thermo-optic coefficient characterizes the rate of RI change with temperature, namely ε=Δn/ΔT, Δn and ΔT are variations of RI and temperature, respectively. Because the length is much larger than the diameter, the thermo-expand coefficient characterizes the rate of microfiber length change with temperature change,  α=(1/L)·(ΔL/ΔT). The thermo-optic coefficient (TOC) and thermo-expand coefficient (TEC) of silica fiber are $-8\times 10^{-6}/{}^{\circ}C$ [93] and $5.6\times 10^{-7}/{}^{\circ}C$ [94], respectively. The temperature sensitivity can be denoted as follows [42]:

(9) $$S_{T}=\frac{\partial \lambda_{N}}{\partial T}=\frac{\lambda_{N}\left(\partial \left(\Delta n_{eff}\right)/\partial T+\alpha_{silica}\Delta n_{eff}\right)}{n_{g}^{even}-n_{g}^{odd}},$$

where $\alpha_{silica}$ is the TEC of silica, and $\Delta n_{eff}$ is the the effective RI difference between two supermodes. The denominator $n_{g}^{even}-n_{g}^{odd}$ is the group-effective RI difference of two supermodes, which is determined by the MFC itself [42].

The temperature-sensing performance of bare MFC is reported in References [23,24,55], and a large dynamic maximum with relatively lower sensitivity has been exhibited. To achieve temperature sensing with high precision, the functional materials with higher TOCs are packaged or sealed outside the MFC. The TOC and TEC of MFC are negligible to the high TOCs of temperature-sensitive materials, so the latter dominants the temperature-sensing process. The small temperature variation will lead to the change of RI of materials, which act as the external medium of MFC. Organic materials are chosen in most of the previous literature [37,42,43,57,58], owing to their excellent thermo-optic property, physicochemical stability, and low cost. The solid materials, such as PDMS, are often utilized to seal the MFC in the form of a thin layer [42]. Liquid materials, like isopropanol, are generally packaged in a tiny vessel, such as a capillary [58]. 

Zhao et al. reported a temperature sensor by packaging the MFC with a waist diameter of 2.2 μm into an isopropanol sealed capillary [43]. A high sensitivity of −5.89 nm/°C has been achieved owing to the high TOC ($\varepsilon =-4.5\times 10^{-4}/{}^{\circ}C$) of isopropanol; the spectrum dip exhibits a remarkable blueshift as temperature increases. This research group utilizes a temperature-controlled water-bath chamber to avoid the disturbances of airflow in the conventional thermostat. Jiang et al. fabricated a Teflon-capillary-encapsulated MFC filled with RI matching liquids to enhance the sensitivity of temperature sensing [58]. When the external temperature changes, the volume of the closed Teflon capillary has a small variation due to the thermo-expand effect, which furtherly leads to an RI variation of filled liquid under the photo-elastic effect. In the process of temperature change, RI variation of liquid dominated by the pressure change rather than the direct temperature change. By combining thermo-expand effect with the photo-elastic effect, the temperature-sensing performance enhances to the maximum. Furtherly, by exploiting a tunable laser source, a photo amplifier, and an oscilloscope, the response time is measured by rapidly changing the ambient temperature. The response time of 243 ms has been recorded by tracking the intensity change of the detector.

Due to the mobility of liquid-sensitive materials, the necessary encapsulation of the sensors results in inconvenience. Compared with liquid-sensitive materials, the solid-sensitive materials can not only serve as temperature transducer, but also enhance the robustness and stability of the sensing system. For example, PDMS, a kind of organosilicon polymer, with merits of low cost, nontoxicity, high TOC ($\varepsilon =-4.5\times 10^{-4}/{}^{\circ}C$), and low RI, is extremely suitable for temperature sensing in microfiber-based sensors. Li et al. utilized PDMS-sealed MFC to monitor environmental temperature change [42]; by tracking the dips near the dispersion turning point, the sensitivity up to 16.78 nm/°C was achieved in the detection range of 26 to 28.5 °C. Besides this, the literature provides simultaneous measurements of multiparameters, including temperature, by utilizing different sensing materials, such as silica gel [57], polyimide [37], and MoS_2_ [31,66].

#### 3.1.3. Humidity Sensors

Humidity, a physical quantity that characterizes the content of water vapor in a gaseous environment, is of great importance in biomedicine industry, construction industry, aerospace industry, et cetera. MFCs may be used to monitor the relative humidity with cooperation of hydrophilic materials. In existing works of MFC humidity sensors, the hydrophilic materials include polyethylene oxide (PEO) [61], silica gel [57], polyvinyl alcohol (PVA) [64], and MoS_2_ [31,66].

**Figure 3:**
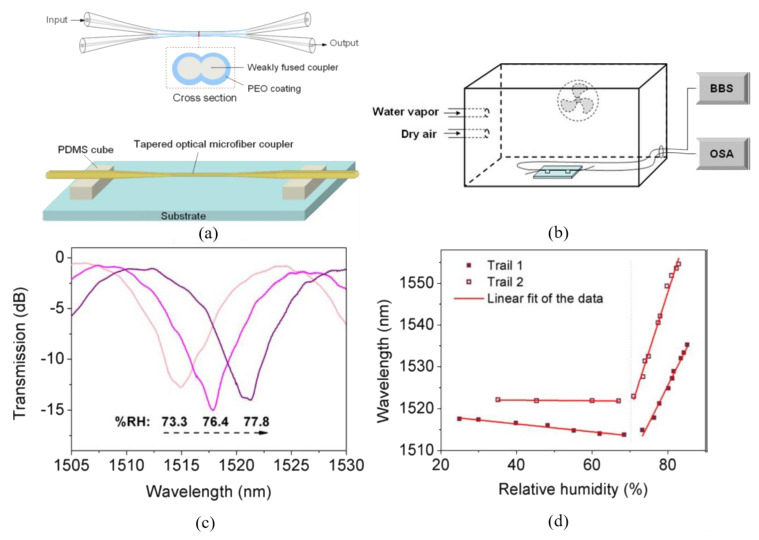
(**a**) Schematic of the polyethylene oxide (PEO) coated MFC. (**b**) Schematic diagram of the experimental setup for humidity sensing. (**c**) Dip wavelength shift of transmission spectrum in the relative humidity range from 73.3% to 77.8%. (**d**) Dip wavelength versus RH for the two samples. Copyright (2015) from Reference [61].

The schematic of an MFC-based humidity sensor fabricated by Bo et al. [61] is shown in Figure 3a. The PEO layer is deposited on the surface of the coupler, and then the coupler is fixed on two PDMS-made cubes with the sensing region suspended in the air. The researchers fabricate two identical MFCs (sample 1 and sample 2) to accomplish the sensing experiment in a humidity-controllable chamber, as described in Figure 3b. By tracking the dip wavelengths at different surrounding relative humidity, from 25% to 85%, the sensitivities of two MFCs may be observed explicitly in Figure 3d. It appears that the sensitivities of the sensors are very low in the relative humidity (RH) range of 25% to 70%. However, they increase dramatically in the RH range of 70% to 85%. This phenomenon may be explained by a phase conversion of PEO from semicrystalline to gel around 80% RH. The RI of the PEO layer decreases drastically at the phase-converting point, due to the fact that density reduction of PEO results from an increase in swelling of the PEO as its water content increases. Utilizing this unique principle, the PEO coated MFC serves as a high-sensitivity humidity sensor in the RH range from 70% to 85%. Figure 3c shows the transmission spectra of sample 1 at the RH of 73.3%, 76.4%, and 77.8%, respectively. The redshift of the dip is observed as the RH increases. The sensitivities of sample 1 and sample 2 are 1.71 and 2.74 nm/%RH in the range of 70% to 85%, respectively.

**Figure 4:**
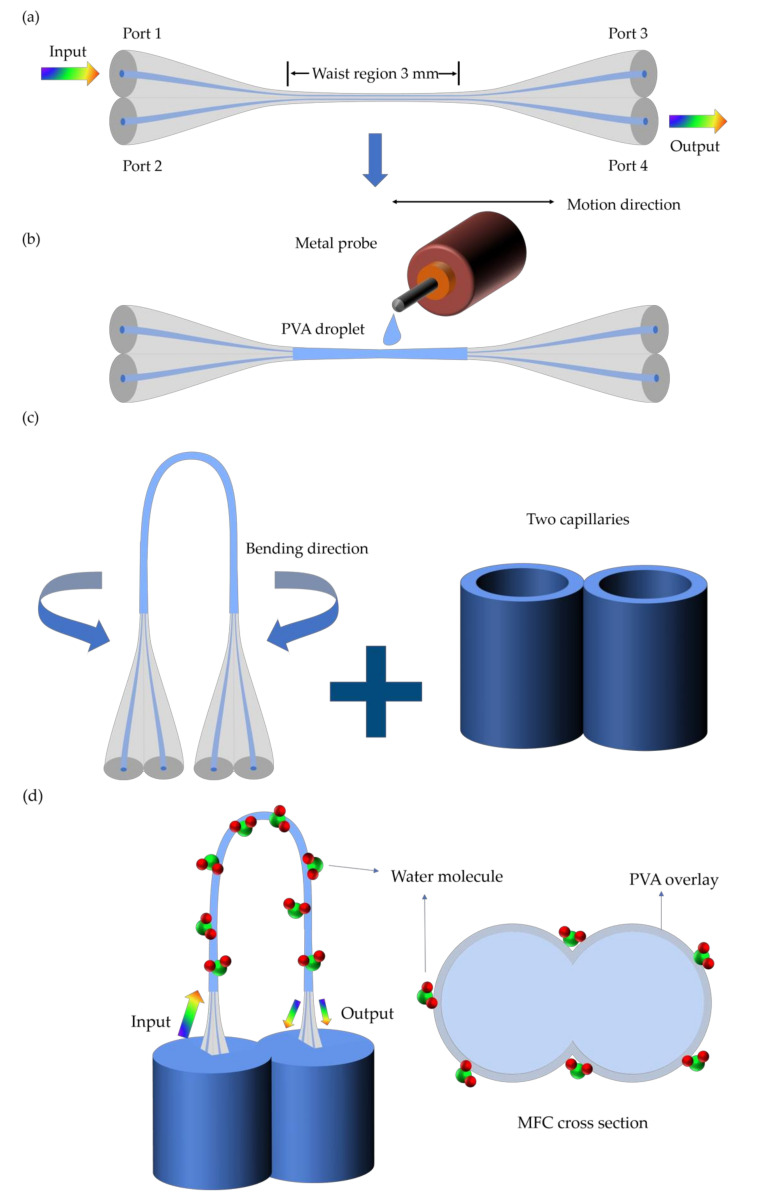
Schematic diagram of (**a**) the MFC and (**b**) PVA-coating technique. (**c**) U-shaped MFC and two capillaries. (**d**) The humidity-sensing process of PVA-coated U-shaped MFC.

Zhao et al. developed a probe-type relative humidity sensor by coating the U-shaped MFC with a PVA film [64]. The structure is novel, and the width of the probe is less than 1 mm, which facilitates potential practical applications in narrow places such as pipes or Petri dishes. The PVA film is widely extended in the field of detection and control of humidity, owing to its perfect performance of absorbing water vapor. After the absorption of water vapor, the volume of PVA film changes drastically and an RI change is induced. To fabricate the U-shaped MFC-based humidity sensor, the preparation processes are briefly shown in Figure 4.

**Figure 5:**
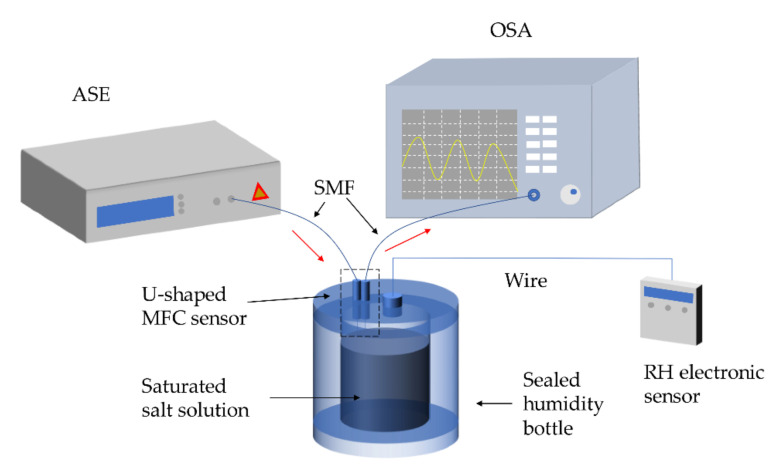
Experiment setup of the proposed sensor.

The MFC is fabricated by the heating-and-pulling method. Then the PVA solution (20 wt%) is made by dispersing the PVA granules into ultrapure water, under the temperature of 80 °C, for 50 min, dropping it uniformly on the waist region of the MFC. After drying for 20 h at room temperature, it is fixed in a U-shape. The two capillaries are used to protect and fixed the MFC. As shown in Figure 5, the RH electronic sensor is used to calibrate the RH and temperature in the sealed the humidity bottle. Slightly different from the experimental setup of Reference [61], this work places the sealed bottles with the salt solutions to meet the requirement of the humidity environment. By recording the corresponding transmission spectra and RH value for each salt solution, the sensitivity for the humidity of this sensor per case is obtained. The highest sensitivity of the U-shaped MFC humidity sensors with a waist diameter of 5.7 μm and waist length of 3 mm reaches 318.1 pm/%RH with good linearity in the wide RH range of 15% to 85% RH. Meanwhile, a low-temperature cross-sensitivity of 13.9 pm/°C from 20 to 50 °C was investigated in this work. Sun et al. [57] and Bai et al. [31,66] realized simultaneous measurement of humidity and temperature by coating the MFC with silica gel and MoS_2_. The sensitivities of 1.6 nm/% RH in the range from 70% to 86% RH, 0.55 nm/°C in the range from 20 to 40 °C, 176.6 pm/%RH in the range from 60.6% to 78.6% RH, and −123.5 pm/°C in the range from 20 to 80 °C were obtained, respectively.

#### 3.1.4. Magnetic Field Sensors

**Figure 6:**
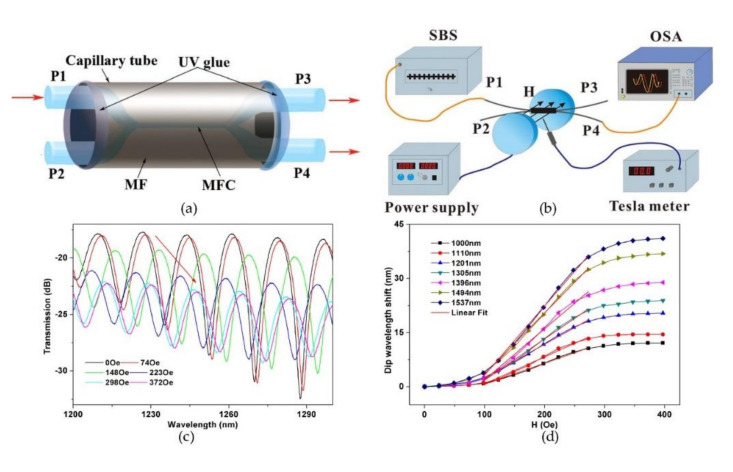
(**a**) Schematic of the MFC-based magnetic field sensor with MF as the surrounding. (**b**) Schematic of the experimental measurement setup. (**c**)Transmission spectral responses to the magnetic field strength. (**d**) Dip wavelength shift as a function of magnetic field strength. Copyright (2015) from Reference [67].

Magnetic field sensors have been used to detect the intensity of the magnetic field and have developed rapidly in recent years. Magnetic field sensors based on MFC may perhaps be categorized into RI-induced and shape-induced. RI-induced magnetic sensors rely on the sensing materials perhaps as a response to ambient magnetic change; shape-induced magnetic sensors depend on the deformation of MFC under physical force. For the first situation, magnetic fluid (MF) is often employed to package the MFC. MF is a kind of functionalized material whose RI varies significantly as the magnetic field intensity change and is generally made by dispersing the magnetic particles with a diameter typically less than 10 nm into solution. With the aid of MF, several highly sensitive magnetic field sensors based on MFC have been investigated. In the same year as Reference [68], Luo et al. proposed a magnetic-field sensor by immersing the MFC with a fiber diameter of 1.8 μm and waist length of 10 mm into the MF environment and well packed in a capillary tube [67]. In the experimental setup, the sensor was placed between two poles of an electromagnet, which can generate a uniform magnetic field with uniformity of larger than 99.9% within the sensing region, and a Tesla meter was used to calibrate the intensity of the magnetic field. The schematics of the sensor and experiment setup are in Figure 6a,b.

By regularly adjusting the intensity of the magnetic field, the different transmission spectra are obtained in Figure 6c. As shown in Figure 6d, the response of wavelength shift for different dips is distinct. The highest sensitivity of 191.8 pm/Oe has been achieved by tracking the dip around 1537 nm. The linearity under the situations of high and low intensity perhaps explained that the magnetic-field-dependent RI of MF satisfied the Langevin-like function. Nevertheless, the linearity in the range from 100 to 250 Oe exhibits high performances.

In 2017, Wei et al. utilized a section of PMF to connect the two output ports of the MFC to form a Sagnac loop [30]. The MFC was immersed in the MF made by Fe_3_O_4_ particles and encapsulated in a PDMS container. The MFC performs well as an interferometer and also as a beam splitter; the output light in port 3 and port 4 counter-propagated in the section of PMF and then recombined at the MFC and experienced a phase difference due to the birefringence of PMF. The Vernier effect induced by the combination of MFC and PMF enhance the sensitivity. As a result, the high sensitivity of −488 pm/mT in the range from 0 to 200 mT achieved an MFC diameter of 2.6 μm and a PMF length of 20 cm.

For the second situation, the MFC was deformed by a magnetic-field-induced physical force. Yan et al. proposed a magnetic field and electric current sensor by bonding an MFC to an aluminum (Al) wire [68]. The detection sensitivity is directly related to the distortion of Al wire from the Lorentz force induced by the magnetic field or the thermo-expand effect induced by electric current.

#### 3.1.5. Other Physical Sensors

In this category, we briefly discuss the various potential sensors that includes twist sensors [27,51], micro-force sensors [50], strain sensors [53], ultrasound sensors [95,96,97,98], vibration sensors [52], current sensors [68], micro-displacement sensors [99], and fluid velocity sensors [70].

Twist sensors based on MFC loop may be realized by either monitoring the coupling ratio [51], or dip wavelength and intensity [27]. In Reference [27], the input and output pigtails of the MFC are fixed by a clamp, and the Sagnac loop is fixed to a rotator. The twist is applied on the MFC by using a rotator, and the transmission spectrum for each twist angle is recorded. The intensity, as well as dip wavelength, is dependent on the twist angle of waist region; sensitivities of 0.9 nm/° and 0.16 dB/° were obtained to confirm the MFC loop has good potential for application in the structural health monitoring and other areas as a twist sensor. The micro-force sensor based on MFC loop has also been proposed by this group [50]; instead of twisting the coupling region, a force in the axial direction is applied on the waist region when the translation stages fixing the pigtails move opposite. Under the axial tension, the lengths of both waist region and transition regions vary, leading to a coupling status change. The proposed micro-force sensor had a high sensitivity of ~3754 nm/N, which is three orders of magnitude larger than traditional optical fiber force sensors, and the detection limit is as low as ~1.6 µN.

For strain sensing, Zhao et al. proposed a sensor formed by utilizing an MZI to connect the MFC and a Sagnac loop [53]. When the axial strain has applied to the coupling region of MFC by two electrically controlled translation stages, the waist length changes slightly. The MZI used offers a wide operating range, as demonstrated by the strain sensor that combined the high sensitivity of MFC and large dynamic range of MZI. The strain-sensing tests show that the sensitivity was −9.3 pm/με in the range of 0–1000 με.

In 2004, Chen et al. fabricated an ultrasonic sensor based on MFC by housing and suspending the MFC within a V-grooved rectangular silica substrate [95]. An acoustic wave is excited in the coupling region when an ultrasonic field is applied to the fiber sensor at one end; as a consequence, the coupling ratio within the fiber coupler alters due to the change in effective strain field in this region. By analyzing the variation of coupling ratio, the information of amplitude and frequency of ultrasound was obtained. The principle of vibration sensor in Reference [52] is similar to this work. Wang et al. put a multimode MFC on an aluminum foil, taking the advantage that multimode fiber is more sensitive to bending due to the characteristics of a larger core and multiple modes. The oscillation of output power results from the vibration of aluminum foil. By analyzing the time domain waveform and fast Fourier transform frequency domain waveform, the MFC-based sensing element was used to detect ultrasound. In recent years, MFC-based ultrasonic sensors have regained interest from researchers; the investigation of the essential response for MFC to ultrasound is discussed in References [96,97].

**Figure 7:**
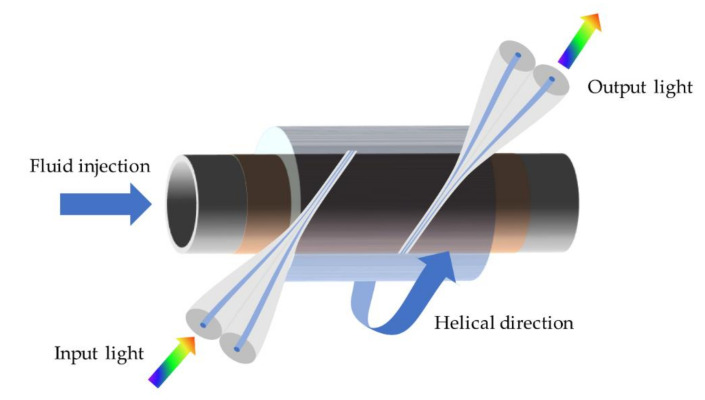
Schematic diagram of the MFC-based microfluidic flowmeter.

Microfluidic flowmeters perform a vital role in molecule detection, cell sorting, and counting. In 2016, Yan et al. reported a novel microfluidic flowmeter based on a helical MFC and a gold-coated glass capillary [70]. The schematic is shown in Figure 7. The MFC is wrapped around the capillary. When the evanescent field is absorbed by the gold film, the heat is generated, and the MFC is warmed. When the microfluid flows through the capillary, heat is taken away, and the temperature of the MFC decreases. As a consequence of temperature change, the dip wavelength of MFC would shift in transmission spectra of output ports. The intensity of dip shift corresponds to the different flow velocities; the device works as a “hot-wire” microfluidic flowmeter. 

Owing to the peculiarity of MFC-based structures, numerous investigations for simultaneous measurement of multiple parameters have been carried out. Here, the multiparameter sensors based on MFC are summarized and compared in Table 3.

For simultaneous measurement of multiparameter, there are two ways to represent the sensitivities for different parameters. The first method has to monitor the various target parameters using the various optical feature demodulation. For example, in Reference [32], the dip wavelength is sensitive to RI change but slightly responsive to temperature change, as the intensity of the transmission spectrum is sensitive to temperature change but insensitive to RI change. The sensitivities for temperature and RI can be valued by the intense and dip wavelength, respectively. The other method is the matrix method. We assure that the selected spectral features (usually preselected dips) vary linearly with ambient measurands. For each particular parameter, each selected feature has a particular sensitivity. We can solve the matrix equation $\left[\begin{array}{c} \Delta \lambda_{1}\\ \Delta \lambda_{2} \end{array}\right]=\left[\begin{array}{cc} S_{\lambda 1-P1} & S_{\lambda 1-P2}\\ S_{\lambda 2-P1} & S_{\lambda 2-P2} \end{array}\right]\left[\begin{array}{c} \Delta P_{1}\\ \Delta P_{2} \end{array}\right]$ to obtain sensitivities of different spectral dips for different sensing objects. The $\Delta \lambda_{i}$ is the wavelength shift of interference dip *i*; $\Delta P_{j}$ is the variation of objective sensing parameter *j*, where i,j=1,2 The $S_{\lambda i-Pj}$ represents the sensitivity for dip *i* to the parameter *j*. As long as the determinant of matrix K is nonzero, i.e., different observed features possess different sensitivities to individual parameters, this matrix might be solved.

### 3.2. Chemical Sensors and Biosensors

Based on the excellent characteristics of light–matter interaction, MFC witnessed as a potential sensing platform for biochemical sensing applications. The targeted molecule might be detected by monitoring the changes of optical transduction mechanisms, such as RI and light absorption, since the biochemical molecule exemplifies capable of binding to the functionalized surface of MFC. Here we review the successful investigations of MFC-based chemical sensors and biosensors.

#### 3.2.1. Chemical Sensors

Chemical composition is crucial in many application scenarios, such as food safety and environmental pollution. MFC-based chemical sensors are investigated to detect the chemical composition in the local environment. Sun et al. reported an ammonia gas sensor based on a silica gel coated MFC with the highest measurement sensitivity of 2.23 nm/ppb (part per billion) and the resolution of 5 ppb [62]. For the proposed MFC with the diameter of 3 µm and 90 nm–thick silica gel coating, the dip wavelength blueshifts along with the increase of ammonia gas concentration, and the response and recovery times are ~50 and ~35 s. One year later, this group additionally realized the methanol and ethanol simultaneous measurement by utilizing a layer mixture of Nile red immobilized sol–gel silica, with the combination of tapered small-core single-mode fiber and MFC [73]. For those gel-coated MFCs, the gel is immobilized on the surface of microfiber by the dip-coating method. For this method, a motor-controlled translation stage is employed to pass a drop of the gel solution through the fiber surface; a single layer of coating is formed after each operation. Therefore, the thickness of gel coating can be well controlled.

**Figure 8:**
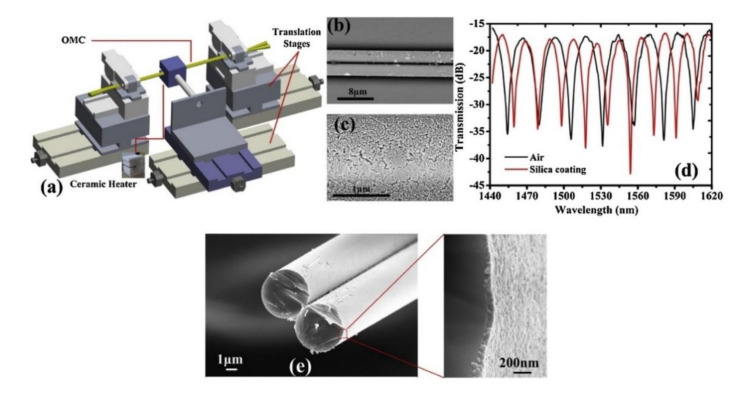
(**a**) The fabrication setup of MFCs; (**b**) the fabricated MFC sensing unit; (**c**) the mesoporous silica coating on the surface of the MFC; (**d**) transmission spectral responses of the MFC before and after it is coated with mesoporous silica layer; (**e**) the cross-section of the fabricated MFC sensing unit. Copyright (2019) from Reference [74].

**Figure 9:**
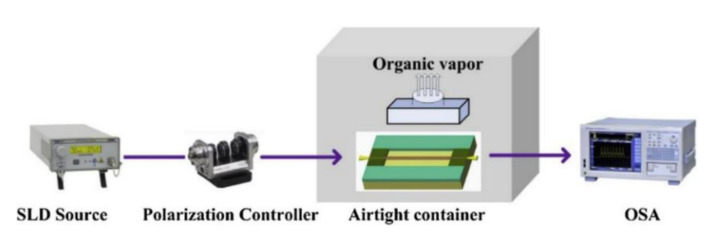
The experimental setup for AMC sensing. Copyright (2019) from Reference [74].

In 2019, Zu et al. proposed a chemical sensor based on MFC loop to detect the concentration of chloride ions by tracking the dip wavelength shift in chloride ion solutions of different concentrations [26]. However, the selectivity to chloride ion has not been established. Selectivity is an essential evaluation factor for a chemical sensor and shows the possibility of crosstalk with other foreign substances. Zhou’s group propose an MFC sensor with a mesoporous silica coating to realize the the detection of airborne molecular contaminants (AMCs) [74]. With the help of mesoporous silica, the absorption ability to AMCs has been enhanced. The fabrication process and sensing experiment setup are described in Figure 8 and Figure 9, respectively.

The MFC sensor has been fabricated by the heating-and-pulling technology, with the mesoporous silica layer coated on the surface of the waist region. The waist diameter and length were 6 µm and 7 mm, respectively. The thickness of the layer was 100 nm. After coating, the transmission spectrum shifted slightly, due to the RI change of the waist surface. Then, the MFC-based sensor was well packaged, to avoid its frangibility and external flowing disturbance, which is shown in Figure 9. The sensor and Simethicone were placed in an airtight container. When the indoor temperature increased, the Simethicone caused outgassing, to change the concentration of contaminants. However, the transmission spectrum remains influenced by the temperature increase and gas pressure change. The total dip wavelength shift reasonably is expressed as $\Delta \lambda =\Delta \lambda_{t}+\Delta \lambda_{p}+\Delta \lambda_{c}$ where $\Delta \lambda_{t}$, $\Delta \lambda_{p},$ and $\Delta \lambda_{c}$ represents wavelength shift induced by the change of temperature, increase of pressure, and bonding of AMCs, respectively. To obviate the influence of temperature and pressure induced wavelength change, the researchers measure the sensitivity to temperature in the thermostat in the range of 35 to 70 °C and calculate the pressure change by the ideal gas equation. It has been emphasized that the $\Delta \lambda_{t}$ and $\Delta \lambda_{p}$ are 9.1 nm and 24.5 pm in the experimental range of 35 to 70 °C, respectively. By monitoring the total dip wavelength shifts as temperature increases, the maximum sensitivity of 0.541 nm/(mg/m^3^) has been achieved, with the permissible detection limit of 36.7 µg/(mg/m^3^).

#### 3.2.2. Label-Free Biological Sensors

With the excellent characteristics of the evanescent field, MFC is of great potential to be developed as a label-free biological sensor which exhibits high sensitivity, low limit of detection, and real-time feedback. Label-free sensors are very appealing, owing to the lack of a process of labeling, which is too complex to be handled. Due to the RI dependence of evanescent field and incompatibility of silica, biocompatible functionalized materials have been used to offer an environment for bio-interaction in the surface of MFC. Here the layer-by-layer self-assembly technique functions intensively used to functionalize the surface of optical fiber with selective reactant therefore enhance the biosensing performances [100]. The receptor molecules are immobilized on the surface of functionalized materials; when the analyte molecules have bonded with a receptor molecule, the RI of microfiber surface changes, thereby inducing a dip wavelength shift or coupling ratio shift. For label-free biosensing, the most important valuation factors are sensitivity, the limit of detection, and specificity. MFC deals with the requirements of high sensitivity and low limit of detection, and high specificity may be realized with the aid of the layer-by-layer self-assembly technique. In the existing literature, the best result of MFC-based biological sensors reaches the limit of detection level of fg/mL [77]. The MFC-based label-free biological sensors are used to detect biomolecules, including cardiac troponin T (cTnT) [80], DNA [78,79], Immunoglobulin G (IgG) [81], streptavidin [49], fibrinogen [75], and cardiac troponin I (cTnI) [77]. Hither, a discussion of these biosensors, in detail, with comparison and evaluation, is presented.

In 2002, the first MFC-based biosensor for protein detection was proposed by Michael Henning from Veridian Corporation [81]. Five years later, Tazawa et al. experimentally demonstrated the MFC-based biosensor for protein detection by intensity demodulation [49], by immobilizing biotin on the coupler surface via amination treatment.

**Figure 10:**
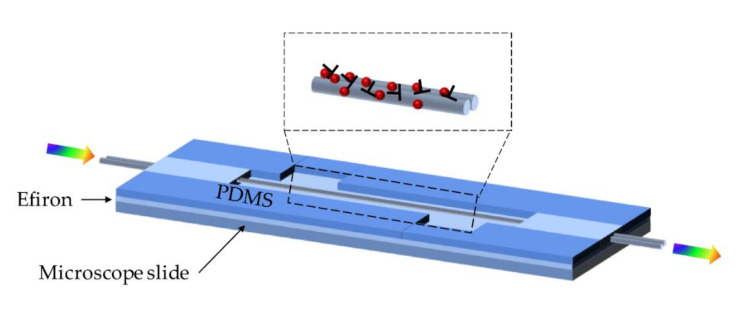
Schematic diagram of the embedded MFC and the experimental setup.

Ismaeel et al. designed and fabricated an MFC-based multiport micro-coil resonator for DNA detection [78,79]. Forming the waist of the MFC into a coil resonator not only increases the stability of the sensor but also makes it ideal for small concentration detection. Due to the subwavelength diameter of the waist region, the MFC was more fragile than other OFSs, so the encapsulation is necessary. In 2014, Bo et al. proposed an MFC-based label-free immunosensor [75]. The sensor typifies well embedded in a low-RI polymer, which enhances the robustness and stability of the sensor. The schematic of the immunosensor is shown in Figure 10.

The fabricated MFC has a waist diameter of 4 µm and two transition regions of 13 mm on each side. For the encapsulation process, a thin layer of the UV curable polymer was employed to cover the silica slide, and then two blocks of PDMS were utilized to create an open-top channel. The MFC remained placed into the channel; UV curable Efiron polymer was employed to block two ends of the channel and the MFC. Because fibrinogen molecule has excellent immobilizing properties on silica surfaces, it is not necessary to silanize the MFC. After human fibrinogen turns immobilized on the surface of the sensor, the sensing area is fully covered with 0.2 mL phosphate-buffered saline (PBS), and the transmission spectrum is found recorded as the baseline, the sensing experiment is carried out by replacing the PBS with different rabbit anti-fibrinogen solution in PBS. In the detection range from 0 to 100 µg/mL, the spectral shifts nearly 10 nm. However, the estimation of specificity was not shown in this work.

For the immobilization of bio-receptor, physical absorption method and covalent coupling method were employed in previous works for a specific label-free MFC biosensor. For physical absorption method, the biomolecules are absorbed on the fiber surface by physical forces, such as polar bond, van der Waals force, hydrogen bond, and so on. As it is a kind of physical absorption with merits of easy operation and less influence on biomolecule, the treatment of fiber surface is not needed, which is established in Reference [75]. For covalent coupling method, the fiber surface is treated to immobilize the bio-transducer. Then the bio-receptor is coupled to the bio-transducer, to realize specific recognition of analyte. In this way, the bio-activity of biomolecules maybe affected by the reaction between the groups. The bio-transducer was immobilized by a layer-by-layer self-assembly method in previous works [77,80].

**Figure 11:**
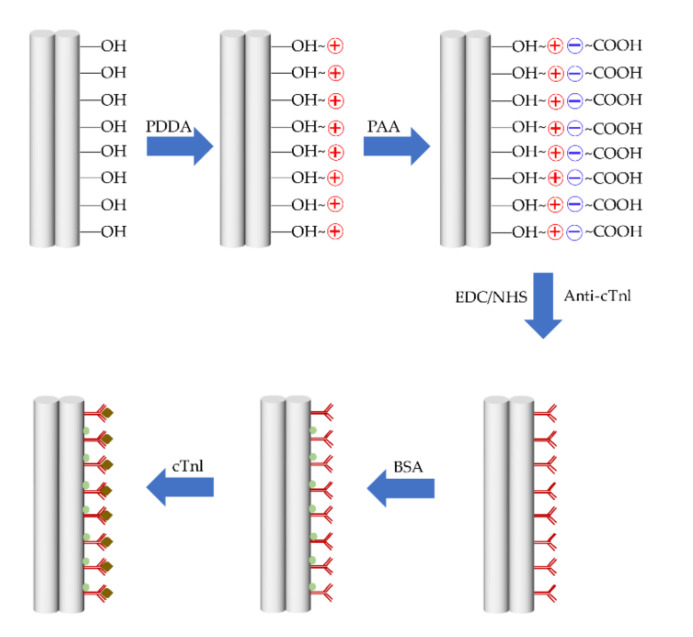
Schematic diagram of the process of waist surface modification and antibody immobilization.

In References [77,78], the layer-by-layer self-assembly method was introduced to enhance the specificity and stability. This method corresponds to a nanofabrication technique to obtain a polyelectrolyte multilayer by immersing the fiber into aqueous polyelectrolyte solutions with an opposite electric charge under the electrostatic force between adjacent layers [100]. This technique remains widely used in optical fiber chemical and biological sensors, combining the optical fiber technology with nanotechnology [101,102,103]. For example, the process of waist surface modification and antibody immobilization in Reference [77] is shown in Figure 11.

Firstly, the sample was cleaned up with deionized H_2_O and then immersed in the KOH solution (0.1 M) for 10 min, to create hydroxyl (-OH) groups on the surface. Then distilled water was used to flush the redundant KOH molecule. Next the 2 mg/mL PDDA (poly-diallyldimethylammonium chloride, positively charged) and 2 mg/mL PAA (poly-acrylic acid, negatively charged) were incubated alternately, for 30 min, on the fiber surface to create a protein-compatible environment. To facilitate capture protein binding, the sample has been immersed in the solution of 50 mM NHS (N-Hydroxysuccinimide) and 200 mM EDC (1-(3-Dimethylaminopropyl)-3-ethalcarbodimide hydrochloride) in water for 30 min to activate the carboxyl group to form reactive NHS. The most important step after a blocking solution (3% BSA (bovine serum albumin) in PBS) represents incubation to block the non-specific binding point. This step ensures that the RI change merges as only induced by antibody-antigen bonding. After that, the cTnI antibody becomes immobilized to the waist surface by incubating the fiber with an antibody solution (100 µg/mL) for 1 h. Then the functionalized biosensor represents ready-to-detect cTnI with concentrations of 2–10 fg/mL. The interaction between antibody and antigen modifies the RI of the functionalized materials which act as the surrounding of MFC. The experiment results show that the detection limit of the biosensor is 2 fg/mL, which runs to the best result of all kinds of fiber optic cTnI biosensors so far.

**Figure 12:**
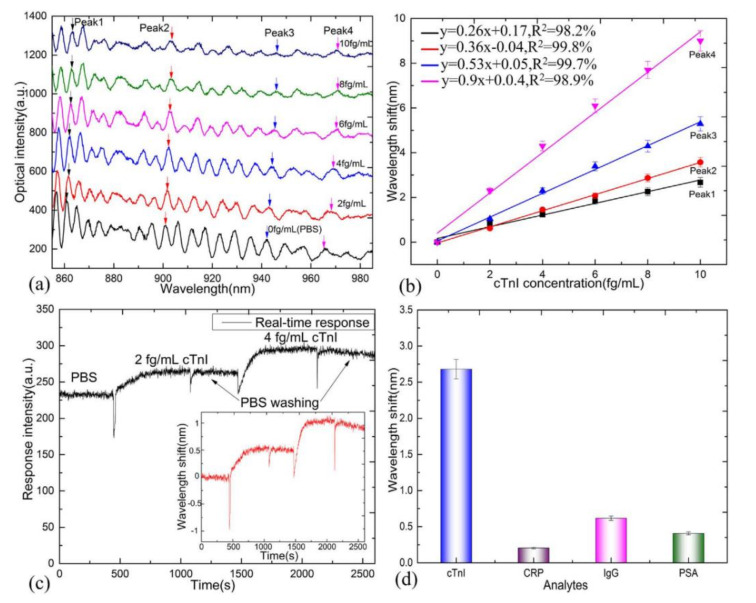
(**a**) Transmission spectral responses near the turning point of effective group index difference (the spectra are offset for clarity). (**b**) Peak wavelength shift with different concentrations of cTnI (PBS, 2, 4, 6, 8, and 10 fg/mL, respectively). (**c**) Real-time response transmission spectrum with PBS, 2 and 4 fg/mL. Inset: corresponding wavelength shift. (**d**) Measured response to cTnI antigen and other non-specific protein (CRP, IgG, and PSA) with the same concentration of 10 fg/mL in PBS buffer. Copyright (2018) from Reference [77].

Besides this, high specificity and fast response time were also investigated in the experiment, as depicted in Figure 12. The better specificity turns out to be proved by monitoring the spectral response in the different kinds of solution. For the specially targeted molecule (cTnI), the wavelength shift could reach up to circa 2.6 nm with the concentration of 10 fg/mL in PBS buffer. However, for the non-special protein, the wavelength shift remains much smaller than special protein with the same concentration. The response time is achieved by analyzing the output intensity at wavelength 904 nm. The signal intensity gradually increases during the interaction process, and it reaches a plateau when the antibody and antigen conjugate completely. Utilizing the similar functionalization process, Reference [80] obtains the detection limit of 1 ng/mL for detection of the human cTnT, which persists a competitive result compared with other label-free cTnT sensors.

The stability, regeneration, and reproducibility are key factors for a practical biosensor, especially for MFC-based biosensors since MFC is easily affected by ambient disturbance. In ref. [80], the real time response curve is measured to show the reaction kinetics. The dip wavelength before and after bio-reaction are constant, which shows the stability of the sensor. In Reference [77], the biosensor is utilized for eight cycles to show the stability, reusability and regeneration. After 8 cycles, the sensing performance without significant losses is obtained. Moreover, from the real-time response transmission spectrum shown in Figure 12c, the stability is provided by valuing the response intensity before and after bio-reaction. The reproducibility is investigated in Reference [75]. Fifteen MFC samples were fabricated, and the sensing performances for each sample were slightly different. This may have been caused by the limited precision in the fabrication.

For label-free biosensors based on MFC, the fabrication, receptor immobilization and encapsulation are the key points to ensure the stability, sensitivity, and specificity. The future work of MFC-based label-free biosensors may focus on these points, to enhance the sensing performance.

## 4. Conclusions and Prospects

In this review, we systematically reviewed the principle fabrication methods that are the state-of-the-art of MFC-based sensors. MFC-based structures have the potential to be applied in the physical- and biochemical-sensing applications, dues to their compact size, simple fabrication, and low cost. Given the diameter of the subwavelength scale, the intrinsic modal interference represents ultrasensitive to the change of the ambient environment. When sensing the RI, the RI change of surrounding persists detected directly and observed in the transmission spectrum. When force, strain, twist, or vibration is applied on the MFC, there might prove to be a deformation on the coupler waist that induces a corresponding spectral shift. To act as a sensor, the functionalized materials responsive turn different parameter variations to RI variation are always introduced to enhance the sensing performance. Different structures based on MFC have their advantages. For example, the coupler tip may perhaps be used as a reflective sensing probe working in scenarios with limited space, while the coupler loop is significantly more convenient to be integrated with other components. Decreasing the diameter of microfiber could increase the sensing sensitivity. However, for the high sensing entity, the smaller sensing dynamic range may be achieved owing to the restriction of instruments operation window. It remains necessary to balance the two essential aspects. The fundamental working principle allows us to further enhance the sensing performance by utilizing new mechanisms, such as dispersion turning point, Vernier effect, and so on. For the MFC, the waist region is very fragile, owing to the ultrathin diameter. The packaging of the MFC plays a critical role to ensure the sensing performance of the sensor. For some physical sensors discussed in this review, the functionalized materials not only serve as transducers, but also enhance the robustness of the sensors.

In the prospects, new sensing mechanisms, packaging methods, and functionalized materials are open for future research, and various configurations for different sensing applications need to be further investigated. As a microfiber-based structure, MFC has a dilemma which is common in microfiber devices. The vulnerability, lack of stability, and repeatability are serious issues existing in MFC-based sensors. It has become necessary to enhance the robustness, stability, and reproductivity, which are highly determined by the fabrication, surface functionalization, and packaging technique. Furthermore, potentially engineering the novel structures based on MFC for further applications, such as wearable sensors or online health-monitoring devices, is efficient for the field of medicine. As a result of the excellent sensing characteristics, MFC-based sensing platforms for the physical and biochemical applications are promising and will attract continuing research interests.

## Figures and Tables

**Table 1:** Part of the microfiber coupler (MFC)-based sensors for different sensing applications.

Year	Configuration	Detected Parameter	Optical Transducer	Sensitivity	Reference
2011	MFC	RI (refractive index)	None	1125 nm/RIU	[34]
2014	MFC loop	Micro-force	None	3754 nm/N	[50]
2014	MFC loop	Twist	None	0.9 nm/°	[27]
2015	MFC	Relative humidity(RH)	Polyethylene oxide	2.23 nm/%RH	[61]
2015	MFC	Magnetic field	Magnetic fluid (MF)	191.8 pm/Oe	[67]
2016	MFC	Flow rate	None	2.183 nm/(μL/s)	[70]
2017	MFC	Ammonia	Silica gel	2.23 nm/ppm	[62]
2018	MFC	Cardiac troponin I (cTnI)	Antibodies + polyelectrolyte layer	2 fg/mL	[77]
2018	MFC	Temperature	RI matching liquid	5.3 nm/°C	[58]
2019	MFC	Airborne molecular contaminants (AMCs)	Mesoporous silica	0.541 nm/(mg/m^3^)	[74]

**Table 2:** The summary of MFC-based temperature sensors with various temperature-sensitive materials.

Years	Configuration	Sensitive Materials	Sensitivity	Dynamic Range	Reference
2012	MFC tip	None	11.96 pm/°C	Up to 1283 °C	[23]
2012	MFC	None	36.59 pm/°C	Up to 1000 °C	[55]
2018	MFC	None	60 pm/°C	84 to 661 °C	[46]
2008	MFC	Organic–inorganic sol–gel	0.17 nm/°C	−50 to 100 °C	[59]
2008	MFC	Glycerol–water solution	−1.5 nm/°C	22 to 60 °C	[54]
2013	MFC	Liquid crystal	0.7 nm/°C	14 to 70 °C	[56]
2015	MFC	Polyimide	1.17 nm/°C	0 to 35 °C	[37]
2016	MFC	Silica gel	0.55 nm/°C	20 to 40 °C	[57]
2018	MFC	Isopropanol	−5.89 nm/°C	30 to 40 °C	[43]
2018	MFC	Glycerin–water solution	5.3 nm/°C	35 to 45 °C	[58]
2018	MFC	Polydimethylsiloxane(PDMS)	16.78 nm/°C	26 to 28.5 °C	[42]
2019	MFC loop	MoS_2_	−123.5 pm/°C	20 to 80 °C	[31]
2020	MFC	Ethanol	−2.03 nm/°C	25 to 55 °C	[92]

**Table 3:** Summarization and comparison of multiparameter sensors based on MFC.

Year	Structure	Materials	Parameters	Sensitivity	Sensing Range	Literature
2015	MFC	Al wire	Magnetic field	~0.0496 mT^−1^	0–10 mT	[68]
			Electric current	~1.0899 A^−1^	0–0.43 A	
2015	MFC	Polyimide	Temperature	1.17 nm/°C	0–35 °C	[37]
			Salinity	−1.03 nm/‰	5–35‰	
2016	MFC	Silica gel	Humidity	1.6 nm/% RH	70–86%RH	[57]
			Temperature	0.55 nm/°C	20–40 °C	
2019	MFC loop	MoS_2_	Humidity	176.6 pm/%RH	60.6–78.6%RH	[31]
			Temperature	−123.5 pm/°C	20–80 °C	
2019	MFC + PMF loop	None	Temperature	0.88 dB/°C	35–41 °C	[32]
			RI	12,020 nm/RIU	1.3333–1.3341	
2020	MFC loop	None	Temperature	−248.2 pm/°C	∼45 °C	[60]
			Salinity	501.4 pm/‰	∼29.5‰	
			Depth (TSD)	122.6 pm/MPa	∼179 MPa	
